# Coping with personal care and stigma: experiences of persons living with schizophrenia

**DOI:** 10.1186/s12912-022-00891-5

**Published:** 2022-05-06

**Authors:** Isaac Tetteh Commey, Jerry Paul K. Ninnoni, Evelyn Asamoah Ampofo

**Affiliations:** 1 Department of Mental Health, School of Nursing and Midwifery, College of Health and Allied Sciences, University of Cape Coast, PMB, Cape Coast, Ghana; 2 Department of Maternal and Child Health, School of Nursing and Midwifery, College of Health and Allied Sciences, University of Cape Coast, PMB, Cape Coast, Ghana

**Keywords:** Ghana, Personal care, Schizophrenia, Stigma, Coping strategies

## Abstract

Living with a chronic mental condition such as schizophrenia impacts significantly on the individual’s social functioning and activities of daily living. However, there is little data on the experiences of people living with schizophrenia, especially in Ghana regarding personal care and stigma. This study explored qualitatively the experiences of people living with schizophrenia in Southern Ghana. Nine people with schizophrenia were purposively recruited for this study. Data were collected using semi-structured interviews and analysed thematically following a descriptive phenomenological data analysis framework. The study revealed that people with schizophrenia are capable of performing some activities of daily living, such as maintenance of personal and environmental hygiene and medication management. However, some participants narrated their experiences of stigma and thus, resorted to certain strategies such as spirituality, medication adherence and mental fortitude to cope with schizophrenia. In conclusion, it was evident that people with schizophrenia, in their lucid intervals, can undertake various activities of daily living, including personal care, however, living with schizophrenia impacts on psychological well-being enormously, and thus, education, counselling, and client adherence to the treatment may improve quality of life.

## Background

Schizophrenia is a chronic mental illness that affects the person psychosocial and economic well-being with significant burdens on their families [1]. Schizophrenia is characterised by difficulties in social contact in everyday life and limits the individual’s ability to participate in the activities of daily life [2]. Persons living with schizophrenia have reported challenges including stigma and difficulty with community integration that affect their physical and psychological wellbeing. Schizophrenia affects over 21 million people worldwide [3]. Understanding schizophrenia has significant implications for health service planning and delivery [4]. Mental illness, mainly schizophrenia, presents a severe health care problem in many African countries; however, limited information exists that explores how the condition impacts on the individual’s life due to a lack of data and poor infrastructure [5]. Schizophrenia is reported as the most diagnosed mental health condition in Ghana [6].

It is claimed that one of the outstanding manifestations of schizophrenia is a disorder of volition [7], where the individuals find it very challenging to maintain their daily living activities. This contributes to why people with schizophrenia are often seen in tattered clothes with unkempt hair in our communities. Personal care reflects the individuals’ activities of daily living [8]. This may include personal tasks to the individual, such as eliminating, communication, maintaining a safe environment, and mobilising. However, most people with schizophrenia may require support to perform daily living activities [9]. In addition, self-care theory portrays individuals as autonomous and suggests that self-care is enacted to regulate the functioning and maintain the health and well-being of people [10–13]. The ability of a person to perform self-care is affected by essential conditioning factors that include the health state of the individual, development state, sociocultural orientation, health care system, family system, patterns of living, environment, and resources [11]. Personal care becomes a challenge to individuals if the condition is not well controlled, and many of them are cared for by their family members and significant others [12].

Furthermore, studies suggest that people with schizophrenia can engage in daily activities such as washing clothes and utensils, sweeping their compounds, taking their baths, general maintenance of personal hygiene, and cooking for themselves and their families [13]. Other studies argue that people with schizophrenia are also capable of engaging in meaningful jobs and contributing to society [14–17]. However, in sub-Sahara Africa including Ghana, people with schizophrenia face enormous stigma, and as a result, they receive limited support leading to poor physical and psychological wellbeing [18, 19]. There is a widening gap in the caregiving literature in Ghana. It is argued that the service user is the primary source of any information regarding their lived experiences and the best person to define recovery [20–24] however, no published study investigated the experiences of people with schizophrenia regarding personal care and stigma in Ghana.

It is argued that one of the most common variables impacting psychological well-being among people with schizophrenia is stigma [25, 26]. Stigma has widely been reported among people living with schizophrenia [26]. This phenomenon seriously limits and reduces the person to a lower social rank [26]. People with schizophrenia are often seen as different from others and are therefore labelled with negative references, which draws them away from the public and limits community integration [27, 28]. One’s ability to cope with negative evaluations and labelling whilst living with schizophrenia is a crucial indicator of the quality of care. Stigma is known to present at several levels: public stigma; self-stigma; stigma by association; structural stigma as the legitimatization and perpetuation of a stigmatized status by society’s institutions and ideological systems [29]. Following the work of Goffman, stigma has been categorised into three dimensions: i) stereotypes are beliefs about a person according to his/her group membership ii) prejudices are attitudes and affective components felt against a person according to his/her group membership and iii) discrimination is behavioural reactions against a person according to his/her group membership [30].

There is considerable evidence showing that people with schizophrenia have been deprived of context-specific needs and lack a comprehensive assessment of their coping strategies adopted in coping with the condition and the related stigma [31]. Therefore, it is imperative to know the subjective experiences of sufferers who have lived with the condition. This is because, studies that have focused primarily on the stories of people living with schizophrenia on coping with personal care and stigma are lacking in Ghana. Furthermore, there is no clear policy guidelines for the management of people with schizophrenia in Ghana, and people with schizophrenia are often seen wonder about in the neighborhood with no shelter. Therefore, it is unclear what the experiences of people with schizophrenia are in Ghana to inform policies. Therefore, this study seeks to contribute to knowledge by addressing three specific issues comprising the context-specific experiences of personal care, stigma and the coping strategies among individuals living with schizophrenia in southern Ghana.

## Methods

### Study design and population

This exploratory-qualitative study adopted the Husserlian descriptive phenomenological design for the study due to the sensitive nature of the subject matter and the need to break new grounds regarding the phenomena. In addition, descriptive phenomenology is mainly employed in qualitative research when little is known about a phenomenon. It focuses on the lived experiences of people with schizophrenia regarding personal care and stigma. The target population for the study included all persons residing within the Cape Coast Metropolis with schizophrenia who had once been diagnosed and managed at the mainstream psychiatric hospital. The Cape Coast is one of the two regions in Ghana with a public psychiatric hospital known as the Ankaful Psychiatric Hospital. It is the only psychiatric hospital in central Ghana that provides mental health services to persons living with severe mental illness on an outpatient and inpatient basis.

The purposive sampling technique was used to access nine (9) individuals with schizophrenia when it was observed that no significant new information was being gathered from participants regarding the phenomena [32]. Participants for the study included people diagnosed with schizophrenia who were within lucidity (the period between recovery and relapse). Data were collected using a semi-structured interview guide. Study participants were interviewed face to face at designated areas predetermined by the researcher and participants. All participants were contacted through telephone calls to explain the study in detail to them. Each participant who agreed to be part of the study was given a participant’s information and consent form to read and sign prior to the study. This form clearly spelt out the rules of engagement for the study, including the benefits which the study is anticipated to bring to persons living with schizophrenia. Those who had literacy challenge and therefore could not read and sign had the content read to them by the researchers after which they signed or thumb printed. Participants duly read and signed the consent forms willingly. To maintain confidentiality and anonymity, each participant chose a pseudonym during the interview and was used throughout the study. In essence, the names of participants in the manuscript are not the real names of persons who took part of the study.

The date, time and place for the interviews were negotiated with the participants. A period of one month (25^th^ May – 22^nd^ June, 2020) was used for the data collection exercise observing all appropriate Covid-19 preventive protocols, such as social distancing, wearing of nose masks, handwashing and hand sanitising. The study was granted ethical clearance by the Institutional Review Board of the University of Cape Coast (UCCIRB/CHAS/2020/37) after demonstrating how conditions of informed consent, anonymity, privacy and confidentiality will be maintained. Guidelines governing ethical considerations in research and protection of the identities of persons spelt out by the Institutional Review Board of the University of Cape Coast were duly adhered to at each stage of the study.

### Inclusion criteria

Participants who met the following criteria were included in the study:

- Persons lives Cape Coast Metropolis for at least one year and with a diagnosis of schizophrenia.
- The person speaks English fluent or any Akan language.
- Adults aged at least 18 years
- The person can give informed consent.

### Exclusion criteria

Participants who met the following criteria were not included in the study:

- All persons in the Cape Coast Metropolis living with schizophrenia who were experiencing active psychotic signs and symptoms of the condition and unable to consent
- Persons less than 18 years were excluded
- Inability to speak the English language or a Ghanaian language

### Data analysis

**Fig. 1:**
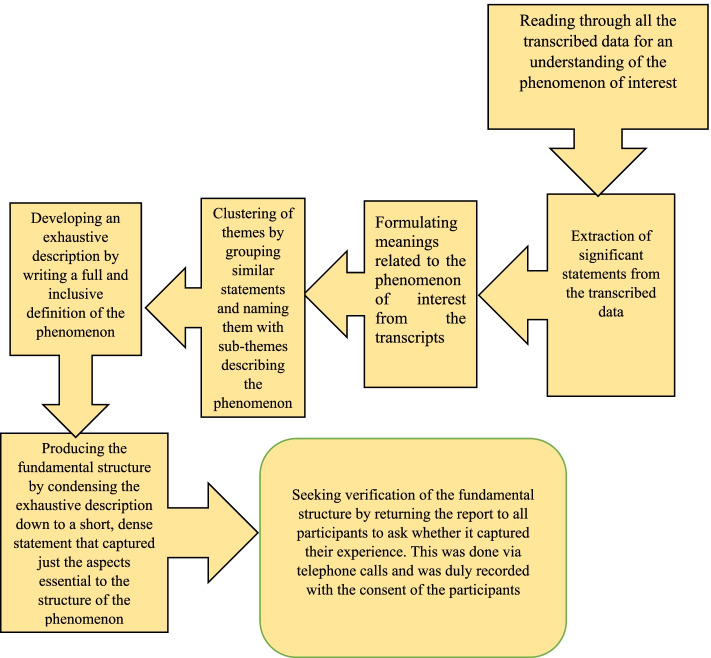
Step by step approach to data analysis

All interviews were transcribed verbatim. The researcher familiarised himself by submerging in the data and carefully reading each transcript thoroughly several times to understand. Significant statements directly relevant to the phenomenon under investigation were identified. Furthermore, meanings pertinent to the phenomenon were then identified. Formulated meanings were then clustered into standard pieces across all participants’ accounts that were significant to the phenomenon under study. A complete and inclusive definition of the phenomenon was written, incorporating all the themes produced under step four. The researcher then condensed the detailed description down to a short, dense statement that captured just those aspects deemed essential to the design of the phenomenon. Finally, verification of the fundamental structure was done. This is where the entire structure statement (report) was returned to all study participants to ask whether it captured their experience. This was done via telephone calls and was duly recorded with the consent of the participants. Earlier steps in the analysis were modified in light of this feedback. Issues other than the phenomenon of concern were not factored into the report because the focus was on the experiences of living with schizophrenia. Figure 1 below demonstrates the step by step approach employed in the analysis of data.

## Results

Key findings of the study have been presented in this section and discussed with existing literature. Table 1 under this section shows the demographic data on study participants.

**Table 1:** Results of demographic data

Demographic Information	Frequency	
**Marital status**		
Married	2	
Single	7	
**Age (in years)**		
20–30	1	
31–40	3	
41and above	5	
**Gender**		
Male	3	
Female	6	
**Religion**		
Christianity	8	
Islamic	1	
**Educational background**		
Junior High	2	
Senior High	4	
Tertiary	3	
**No. of Years with Schizophrenia**		
1–10	2	
11–20	2	
21 and above	5	

It can be noted from the table that, out of nine participants, seven were single while two were married. On the age range of participants, the data revealed that one of the participants was in the age range of 20 -30 years. This was followed by three who were in the age range of 31–40 years. Also, the remaining five of the participants were in the age range of 41 years and above. Concerning the gender of the participants, six were females whilst three were males.

As part of the demographic characteristics of the respondents, the religious affiliation of the respondents was considered. Again, eight of the participants were Christians except one who declared that he was a Muslim. For the educational background of the respondents, two completed junior high school, four senior high school and three had tertiary education. The study also took into consideration the number of years respondents had lived with the diagnosis of schizophrenia. Five of the respondents had experienced the condition for 21 years and above. Two respondents had lived with schizophrenia for 11 to 20 years whilst the remaining two were diagnosed with schizophrenia within a period of 1 to10 years ago.

### Summary of key qualitative findings

Analysis of the interviews data generated two main themes which describe participants experiences regarding schizophrenia. These themes include; personal care; Stigma and coping strategies which include mental fortitude, Spirituality and adherence to medication.

### Personal care: activities of daily living

Study participants expressed their views on sticking to daily living activities to live with the condition. Participants disclosed that having lived with schizophrenia for several years, they have accepted that the condition is part of them and something to live with. They indicated that they could take care of their daily activities such as meeting their personal hygiene needs, nutritional demands, and sleep despite their condition. The account shows that participants could go about their normal activities of daily living without any concerns.

*“……I have been living with this condition for several years without any interference in the discharge of my daily activities both at home and when I go to school to teach. I do my things as expected of every human being. I maintain my home very well before going to school. I teach the children, as usual, interact with colleague staff in the school, and carry out my responsibilities as the head of my family. I enjoy my sleep and always take care of myself very well. It is only when the condition comes that I see some changes. (Terry, 58years; 25*^*th*^ May 2020).

Another participant also added that:

*…... “daily activities have never been my problem. When you came you saw me washing; I just finished cooking for my parents. They are inside eating. I will take my lunch after washing. I enjoy doing house chores. They keep me active and strong” (Favour, 30 years; 28*^*th*^* May 2020).*

### Stigma

 Respondents reported that stigma is one significant negative experience they have had to cope with ever since they were diagnosed with this condition. They indicated that they have been at the receiving end of name-calling, labelling and neglect at the hands of people. Study participants believed that people in their community (public concern) are the ones who stigmatise them. It was clear from participants’ accounts that all these negative experiences did not come from family members. They indicated that the family members did not mistreat them at all. However, people who lived outside their homes were the ones who negatively evaluated them most often. Participants pointed out that stigmatisation is associated with schizophrenia just like any other chronic mental illness, which sufferers cannot avoid once they live with the disease. One of the powerful stories on this subject matter can be found below:

*“…….. this is my major challenge associated with this condition…over the years, I have come to recognise that people don’t understand my condition…… they point fingers at me, call me all sorts of names and say negative things about me. One day, I stopped a car on my way to church, and just when I was about to board the car, one woman around the place quickly ran to inform the driver and the people in the car that I was a mad person, so the driver should not pick me. I had to walk to church that day*. *This sometimes makes me angry, anxious, and sad.” (*Beauty, 44 years; 22^nd^ May 2020).

### Coping strategies

#### Mental fortitude

The data revealed that respondents adopt bold measures in coping with schizophrenia despite the challenges associated with the condition. These measures enable them to maintain some level of resilience. Study participants verbalised that the strategy they adopt most often to cope with their illness is deliberately trying to take their minds off it. In other words, participants could prevent possible schizophrenic relapse by avoiding excessive thinking or worrying about their situation and its associated impact on their living conditions. A participant, for example, believes that accepting his condition and refusing to worry about it is a way to cope. “*The negative things associated with this illness do not worry me. I have come to accept that schizophrenia has become part of me, so I don't bother myself with negative things. The more I think about it; the more my condition gets worse….”* (Godswill, 49 years: 19^th^ June 2020).

### Adherence (medical care)

As part of the maintenance of personal care, respondents affirmed that they adopted some medical measures to help them stay healthy. These participants asserted that one of the major coping strategies had been medication adherence. Participants adhered to the treatment plan at the mental health facilities as part of their care. They explained that failure to comply with the treatment plan results in schizophrenic relapse.

*“… The medication has helped me a lot; it is my food. I do not skip my medication because it has saved my life. Despite the bad side effects associated with the medication at times, I still think it is what keeps me from experiencing a relapse.” (Forgive, 38 years; 9*^*th*^* June 2020).*

### Spiritual well-being

Participants expressed that their belief in God who keep them going. This, according to them, sticking strictly to spiritual principles helps them gain some sense of hope and encouragement and thus prevents them from experiencing a relapse. In other words, their religious faith has been a source of hope in keeping them healthy. They indicated that their faith in God and spiritual activities in their places of fellowship give them some sense of relief and increase their understanding of well-being. Remain resilient.

*“……I am a good Christian. I have faith in God, which has been my source of hope all these years. Prayer meetings and church activities take place at my church to take my mind off this illness and give me some form of relief. I believe I have not suffered from relapse all this while because of my constant church involvement.” (Shallom, 19 years, 20*^*th*^* June 2020)*

*“…. Although I take my medications daily as instructed, I believe Allah is the true healer who can heal me of this condition. Whenever I go to the mosque and meet my fellow Muslim brothers for prayers, I experience great joy and relief. I feel like I don’t have any problem in my life anytime I find myself amid Muslim brothers and sisters.” (Bella, 43years, 22*^*nd*^* June 2020)*

## Discussion

This study explored the experiences of persons living with schizophrenia in Cape Coast, Ghana. It has brought to the fore, personal care experiences, stigma and coping strategies adopted by persons living with schizophrenia. Before the study, there was no empirical literature regarding the subjective experiences of persons with schizophrenia in Cape Coast (Southern Ghana). This study went further to explore personal and subjective issues of concern in the lives of individuals with schizophrenia. It explored critical issues centred on how they have taken care of themselves and coped with schizophrenia-related stigma despite living with this illness.

There is empirical evidence on the coping strategies adopted by care givers of individuals with schizophrenia [33]. However, literature on the coping strategies of individuals who have lived with schizophrenia over a period of time in Ghana is hard to find. Participants in this current study pointed out some personal care experiences whilst living with the condition.

Regarding the maintenance of activities of daily living, the findings showed that participants could do things in their rightful sense just like any ordinary person in society. It also suggests that they can do things independently without necessarily depending on others in discharging duties such as bathing, eating, washing and other household chores. It is not surprising that a previous study supports this assertion. Similar research was conducted in Sweden [34] to describe the engagements in daily activities of people with schizophrenia and revealed that being diagnosed with schizophrenia does not necessarily lead to an impoverished lifestyle. Instead, individuals with schizophrenia could have a normal lifestyle and perform activities such as washing, bathing etc. and even observe personal hygiene. Contrary to an assertion that persons with schizophrenia have certain deficits in their lives that render them unable to carry out activities of daily living at will [35, 36], participants in this study proved that, despite living with schizophrenia, the condition did not interfere with their daily living activities in any way.

Persons with schizophrenia have been at the receiving end of name-calling, insults, and discrimination over the years. There have been some negative comments, assessments, and discrimination attributed to persons living with the diagnosis of schizophrenia by people who come into contact with them. Stigmatisation has been noticed to have characterised the lives of persons with schizophrenia [37–40]. These individuals are highly discriminated against, especially in communities where they live, partly because society perceives them as mentally ill and a threat to the community. Thus, the community does not see the essence of associating with the "mad" people in the community. Another reason people stigmatise these individuals may be due to the symptoms people with schizophrenia exhibit, especially in the relapse stage. Such manifestations scare people, especially those in the catchment area where study participants reside [41]. Hence, most people in the community may consider them highly violent and can even kill people who may get closer to them. Society may permanently stigmatise them [41]. Stigma affects the well-being of people with schizophrenia because it leads to isolation and rejection of these victims.

Schizophrenia is the most stigmatised of all mental conditions because of its perceived dangerous and unpredictable nature [40–42]. As a result of stigma, these persons become angry, anxious, scared of the unknown, and sad. People’s perception of schizophrenia and how they label people with the condition make it very challenging for individuals living with the condition to cope with it, especially when they step out of their homes [42]. They, therefore, respond to these unfortunate situations by using defence mechanisms such as avoidance, denial, and resorting to wishful thinking consistent with previous findings [41]. Contrary to what was found in Croatia on schizophrenia and stigma, which indicated that, mental health nurses and nurses working in other general hospitals do stigmatise persons with schizophrenia [42], study participants in this current study verbalized that, mental health nurses, often get closer to them and encourage them to avoid taking into how people negatively evaluate them in society.

Regarding coping through mental fortitude, study participants with schizophrenia believed that the condition had become part of their daily lives, therefore they do not stress themselves about the negative manifestations associated with the condition. They are able to deal with the challenges associated with the condition by indulging in wishful thinking. This presupposes that, if an individual with schizophrenia does not stress him or herself by thinking excessively about the condition, the possibility of experiencing a relapse is minimal because it is believed that stress as a result of thinking excessively about the condition can lead to frequent relapse. This finding is not different from a similar qualitative study conducted on the coping strategies adopted by individuals with schizophrenia in Great Britain [43]. He also opined that persons with schizophrenia engage in activities that divert their attention from the negative aspects of the condition to stay healthy.

Participants verbalized that; they cope with their condition by strict adherence to regular intake of their prescribed medication. Hence, medication was a decisive factor in protecting individuals from experiencing a schizophrenic relapse. With adequate adherence to medicines, participants with schizophrenia could maintain resilience and feel more comfortable going about their normal daily activities. Participants asserted that, those of them who adhered to medications had a quality of life compared to their counterparts who did not follow strictly their treatment plan. It is documented that patients with schizophrenia cope well with their condition through regular adherence to prescribed medications [44–46]. This, according to them, makes them strong and prevents them from experiencing any relapse [45]. Steady medication adherence helps persons with schizophrenia improve their care and cope with the condition.

Spiritual wellbeing, including participating in religious activities, creates a sense of belonging, enables them to deal with difficult situations, and gives them the strength to move on despite their condition. This may imply that; religion possibly provides positive coping to patients with schizophrenia and subsequently help in recovery. Religious coping was the most common strategy used by people with schizophrenia to cope with daily activities associated with the condition [47–49]. This may be because participants indicated that religious coping enhances self-esteem and reduces adverse effects associated with schizophrenia. Increased self-esteem can be shown to contribute to a positive health outcome. Religious faith also serves as a source of strength for persons with schizophrenia and assures them that they can survive complex events in their life. Participants’ belief in their maker improves their relationship with family and other people in their communities [49]. Persons diagnosed with schizophrenia cope well through religious activities such as exorcism or sacraments, which they believe could restore their mental and physical well-being to normalcy [47]. The implication is that people with schizophrenia will always depend on religion to manage the condition due to the relief or solace they derive from it.

## Conclusions

This study has shed light on how people with schizophrenia live and cope with their personal care and the stigma associated with their conditions in Ghana's resource-constrained setting. Participants in their lucid state lived an everyday life and could maintain daily living activities successfully. Again, study participants verbalised being labelled and seen as different within the communities in which they reside. It was evident in the study that persons with schizophrenia adopt subjective measures that help them to live with the condition and cope with the associated stigma despite the challenges that the situation presents to them. This calls for the need to intensify education to reduce public stigma regarding schizophrenia.

Overall, these findings are not dramatically different from those reported in the literature; however, the support needs of people with schizophrenia may differ from a cultural and spiritual point of view. Africa and, therefore, Ghana is highly religious, and people find solace in religious coping strategies. As patient advocates, nurses also need to appraise their educational programs to address stigma and avoid the increasing trend of societal beliefs regarding persons living with schizophrenia. Positive media representation of people diagnosed with schizophrenia would also go a long way in reducing their opposing expectations of themselves and replacing them with personal strength, hope and aspirations.

## Limitation of the study

Qualitative research is often criticised for lacking generalizability and being too reliant on the subjective interpretations of researchers. Therefore, the results of this study cannot be generalised as the true reflection of all persons living with schizophrenia in the country. However, it was not the researcher’s aim to make generalisations but to understand and describe the experiences of persons living with the diagnosis of schizophrenia in the Cape Coast Metropolis of Ghana.

## Recommendations

Based on the findings of the study, the following recommendations were made:

### Nursing practice

Community psychiatric nurses should continue to intensify their home visits to individuals living with schizophrenia in their catchment area to support clients who have challenges with personal care and stigmatisation.

### Education

The mental health authority (MHA) of Ghana should intensify health education on issues relating to schizophrenia to create awareness on issues affecting the lives of Persons Living with Schizophrenia.

### Policy

There are no clear policy guidelines in Ghana that primarily focuses on the management of schizophrenia in the country. This study, however, recommends that authorities at Cape Coast metro health directorate should establish a counseling centre within its premises to house accredited religious ministers and professional psychologists to meet the needs of clients with schizophrenia and their families.

## Suggestions for further study

Research can be conducted on gender differences in the experience of Persons Living with Schizophrenia to find out if differences exist between males and females living with schizophrenia.

## Data Availability

The raw and analysed data are available with the corresponding author on reasonable request.
